# TAGLN2 is a candidate prognostic biomarker promoting tumorigenesis in human gliomas

**DOI:** 10.1186/s13046-017-0619-9

**Published:** 2017-11-06

**Authors:** Ming-Zhi Han, Ran Xu, Yang-Yang Xu, Xin Zhang, Shi-Lei Ni, Bin Huang, An-Jing Chen, Yu-Zhen Wei, Shuai Wang, Wen-Jie Li, Qing Zhang, Gang Li, Xin-Gang Li, Jian Wang

**Affiliations:** 1 Department of Neurosurgery, Qilu Hospital of Shandong University and Brain Science Research Institute, Shandong University, #107 Wenhua Xi Road, Jinan, 250012 China; 2 Department of Biomedicine, University of Bergen, Jonas Lies vei 91, 5009 Bergen, Norway

**Keywords:** TAGLN2, Glioma, EMT, Cell cycle, TGFβ signaling

## Abstract

**Background:**

Transgelin-2 (TAGLN2) is a member of the calponin family of actin-bundling proteins that is involved in the regulation of cell morphology, motility, and cell transformation. Here, the clinical significance and potential function of TAGLN2 in malignant gliomas were investigated.

**Methods:**

Molecular and clinical data was obtained from The Cancer Genome Atlas (TCGA) database. Gene ontology and pathway analysis was used to predict potential functions of TAGLN2. RNA knockdown was performed using siRNA or lentiviral contructs in U87MG and U251 glioma cell lines. Cells were characterized in vitro or implanted in vivo to generate orthotopic xenografts in order to assess molecular status, cell proliferation/survival, and invasion by Western blotting, flow cytometry, and 3D tumor spheroid invasion assay, respectively.

**Results:**

Increased *TAGLN2* expression was associated with increasing tumor grade (*P* < 0.001), the mesenchymal molecular glioma subtype and worse prognosis in patients (*P* < 0.001). Immunohistochemistry performed with anti-TAGLN2 on an independent cohort of patients (*n* = 46) confirmed these results. Gene silencing of TAGLN2 in U87MG and U251 significantly inhibited invasion and tumor growth in vitro and in vivo*.* Western blot analysis revealed that epithelial-mesenchymal transition (EMT) molecular markers, such as N-cadherin, E-cadherin, and Snail, were regulated in a manner corresponding to suppression of the EMT phenotype in knockdown experiments. Finally, TAGLN2 was induced ~ 2 to 3-fold in U87MG and U251 cells by TGFβ2, which was also elevated in GBM and highly correlated with *TAGLN2* mRNA levels (*P* < 0.001).

**Conclusions:**

Our findings indicate that TAGLN2 exerts a role in promoting the development of human glioma. The regulation and function of TAGLN2 therefore renders it as a candidate molecular target for the treatment of GBM.

**Electronic supplementary material:**

The online version of this article (10.1186/s13046-017-0619-9) contains supplementary material, which is available to authorized users.

## Background

Human gliomas are the most common and deadly type of primary intracranial tumors and account for approximately 80% of all primary brain malignancies. Gliomas are histopathologically classified into four tumor grades (I–IV) according to the World Health Organization (WHO). WHO grade IV or glioblastoma multiforme (GBM) confers the worst prognosis, with a median survival time of merely 12 to 15 months following primary diagnosis [1, 2].

With advancement of gene technology, molecular signatures have become prominent in the classification of gliomas in recent years. Whole-genome analysis of patient cases through The Cancer Genome Atlas (TCGA), revealed a molecular classification scheme for GBM which includes four molecular subtypes: proneural, neural, classical, and mesenchymal. Among these four subtypes, the mesenchymal subtype was distinguished from the others as being particularly aggressive [3–5]. Thus, there is an urgent need for the exploration of novel biomarkers and therapeutic targets for GBM molecularly classified as the mesenchymal subtype.

Transgelin-2 (TAGLN2) is an actin-cross-linking protein containing a calponin homolog (CH) domain, with a molecular weight of 24 kDa. It has been reported that TAGLN2 is involved in the regulation of cell transformation and cell morphology [6, 7]. More recently, the dysregulation of TAGLN2 in a variety of malignant tumor types, including colorectal cancer [8], bladder cancer [9], lung cancer [10], uterine cervical squamous cell carcinoma [11], and breast cancer [12], has been discovered through proteomic analysis, and thus reveals an important role for TAGLN2 in tumor progression. The expression pattern and clinical significance of TAGLN2 in human gliomas, however, have not been determined. Furthermore, it remains unknown as to whether TAGLN2, because of its role in cell transformation and cell morphology, is involved in the regulation of the epithelial-mesenchymal transition (EMT).

Here, we used publicly available datasets to determine the pattern of *TAGLN2* expression in human gliomas, and its relationship with tumor grade, and other clinicopathological indicators and molecular features of gliomas. TAGLN2 function was investigated both in vitro and in vivo as well as potential pathways regulating it. Our findings indicate that TAGLN2 might be a significant prognostic indicator and a potential therapeutic target for human gliomas.

## Methods

### Clinical specimens and databases

Archived paraffin embedded glioma tissues (WHO grades II-IV) were collected from patients (*n* = 46) who underwent surgery in the Department of Neurosurgery, Qilu Hospital of Shandong University. Normal brain tissue samples (*n* = 5) were taken from trauma patients who underwent partial resection of normal brain as decompression treatment for severe head injuries. mRNA expression microarray data and accompanying clinical information for samples in The Cancer Genome Atlas Research Network (*n* = 667; TCGA, http://cancergenome.nih.gov) were used for analysis. Four external independent glioma databases (Rembrandt, CGGA, Gravendeel, and GSE4271) were also mined.

### Immunohistochemistry (IHC)

Sections (4 μm) were obtained from formalin-fixed, paraffin-embedded tissues of different grades of human gliomas. Sections were boiled in sodium citrate buffer (pH 6.0) for antigen retrieval, and endogenous HRP activity was blocked with 3% H_2_O_2_. Slides were blocked with 10% normal goat serum and incubated with primary antibody (mouse anti-TAGLN2 monoclonal antibody, 1:25; Santa Cruz; Dallas, TX, USA) at 4 °C overnight. Signal was visualized using standard protocols with horse radish peroxidase conjugated secondary antibody and 3, 3′-diaminobenzidine (DAB) as the substrate. For negative controls, sections were incubated with normal mouse serum rather than primary antibody. Slides were counterstained with hematoxylin, and representative images were obtained using an Olympus inverted microscope.

### Cell culture

U87MG and U251 human GBM cell lines were purchased from the Culture Collection of the Chinese Academy of Sciences (Shanghai, China), and cultured in Dulbecco’s modified Eagle’s medium (DMEM; Thermo Fisher Scientific; Waltham, MA, USA) supplemented with 10% fetal bovine serum (FBS; Thermo Fisher Scientific, USA). The patient-derived primary GBM cells (GBM#P3, mesenchymal subtype) were kindly provided by Professor Rolf Bjerkvig, Department of Biomedicine, University of Bergen, Norway. GBM#P3 cells were cultured in Neurobasal Medium (Thermo Fisher Scientific; Waltham, MA, USA) containing B27 supplement (20 μL/mL), FGF (20 ng/mL) and EGF (20 ng/mL). Cells were maintained at 37 °C in a humidified chamber containing 5% CO_2_.

### Gene ontology (GO) and Kyoto encyclopedia of genes and genomes (KEGG) analysis

Correlation analysis of *TAGLN2* was performed in gene expression profiles available in the TCGA dataset with Matlab software (https://cn.mathworks.com). To identify biological processes and the KEGG signaling pathways associated with *TAGLN2* expression in gliomas, genes positively and negatively correlated with *TAGLN2* (*P* < 0.01) were analyzed using the DAVID web tool (http://david.abcc.ncifcrf.gov/home.jsp). Association between *TAGLN2* expression and hallmark gene sets from the Molecular Signatures Database (MSigDB) were analyzed using gene set enrichment analysis (GSEA) software (http://software.broadinstitute.org/).

### *TAGLN2* silencing

Small interfering RNA (siRNA) targeting *TAGLN2* were synthesized (GenePharma; Shanghai, China). siRNAs were transfected with Lipofectamine RNAiMAX reagent (Thermo Fisher Scientific; Waltham, MA, USA) according to the manufacturer’s protocol. Stable knockdown of *TAGLN2* in cells was generated using lentiviral transduction of sh-TAGLN2 (Genepharm). Knockdown efficiency was evaluated 48 h after transfection by Western blotting. siRNA sequences (*n* = 2) that generated efficient knockdown are the following: si-TAGLN2#1: 5′-GCAAGAACGUGAUCGGGUU-3′; and si-TAGLN2#2: 5′-UAUGUGAGCUCAUUAAUGC-3′. The second sequence of siRNA was used for the functional assays in vitro.

### Western blotting

Harvested cells were lysed with heat denaturation in RIPA cell lysis buffer. Protein lysates (20 μg) were run on SDS-PAGE, and proteins were transferred to polyvinylidene difluoride (PVDF) membrane. Blots were incubated primary antibodies against TAGLN2 (Santa Cruz); N-cadherin, E-cadherin, β-catenin, Snail, Slug, Twist, p21, p27, CDK2, Survivin, c-Myc, Cyclin D1, CD44, GAPDH (Cell Signaling Technology; Danvers, MA, USA); and FoxM1, Cyclin B1, Smad, p-Smad, CHI3L1 (Abcam; Cambridge, UK). Specific proteins were detected with enhanced chemiluminescence (ECL, Millipore, Bredford, USA). Band density was measured (ImageJ software) and normalized to GAPDH.

### 3D tumor spheroid invasion assay

Glioma spheroids were generated by incubating cells in the spheroid formation matrix for 72 h in a 3D culture qualified 96-well spheroid formation plate. Spheroids with a diameter of >200 mm were embedded into the invasion matrix (Trevigen, Gaithersburg, USA) composed of basement membrane proteins in the 96-well plate. Glioma spheroids were photographed every 24 h under Nikon microscopy. The spheroid at 0 h was used as a reference point for measurement of the area invaded by sprouting cells.

### Immunofluorescence

Transfected cells were fixed with 4% paraformaldehyde for 15 min at room temperature, rinsed with phosphate buffered saline (PBS), permeabilized with 0.4% Triton X-100 for 10 min, and blocked with 10% goat serum for 60 min at room temperature. Coverslips were incubated at 4 °C overnight with anti-N-cadherin and anti-E-cadherin antibody, followed by incubation for 1 h with an Alexa-conjugated secondary antibody. Alexa Fluor® 594 labeled phalloidin (Life Technologies, USA) was used to visualize F-actin in the cytoskeleton, and nuclei were stained with DAPI. Representative images were obtained with a Nikon inverted fluorescence microscope.

### Cell proliferation assay

Cell proliferation was measured using the Cell Counting Kit-8 (CCK-8) according to the manufacturer’s instructions (Dojindo, Kumamoto, Japan). U87MG or U251 (at 2 × 10^3^ cells/well) were incubated in 96-well plates for 24, 48, and 72 h. CCK-8 solution (10 μL) was added to each well, the plates were incubated for 1 h at 37 °C, and absorbance at 450 nm wavelength (OD450) was measured in a Microplate Reader (Bio-Rad). For the EdU assay, cells were incubated with 200 μL of 5-ethynyl-20-deoxyuridine (Ribo-Bio; Guangzhou, China) for 2 h at 37 °C. Cells were fixed in 4% paraformaldehyde for 20 min, permeabilized with 0.4% Triton X-100 for 10 min, and incubated with Apollo® reagent (100 μL) for 30 min. Nuclei were stained with DAPI, and representative images obtained with a Nikon inverted fluorescence microscope.

### Flow cytometry

Cell cycle analysis was performed by determining DNA content with propidium iodide (PI) staining (BD Biosciences; San Jose, CA, USA). Briefly, U87MG and U251 glioma cells were harvested, re-suspended and stained with propidium iodide (PI; BD Biosciences) in the presence of RNase A for 20 min. Apoptosis was evaluated in U87MG and U251 cells with Annexin V-FITC and PI staining (20 min; BD Biosciences). Cells were analyzed using a flow cytometer (BD Biosciences) according to the manufacturer’s instructions.

### Implantations in nude mice

To establish intracranial gliomas, U87MG and U251 cells (1 × 10^6^) were infected with Lenti-si-TAGLN2 or Lenti-Control virus and then implanted stereotactically into the brain of 4-week-old nude mice (SLAC laboratory animal Center; Shanghai, China). Tumor tissues were harvested, formalin-fixed and paraffin-embedded, sectioned (4 μm) and incubated with antibodies against TAGLN2 (Santa Cruz, USA), N-cadherin and Ki-67 (Abcam, UK).

### Statistical analysis

Survival curves were estimated by the Kaplan-Meier method and compared using the log-rank test. The cut-off level was set at the median value of *TAGLN2* expression levels. The expression pattern of *TAGLN2* in different glioma subtypes and the associations of *TAGLN2* with isocitrate dehydrogenase 1 (*IDH1*) mutation, methylation of O-methylguanine-DNA methyltransferase (*MGMT*) promoter, codeletion of 1p/19q, telomerase reverse transcriptase (*TERT*) loss, and alpha thalassemia/mental retardation syndrome X-linked (*ATRX*) mutation were performed using the TCGA dataset. A two-tailed χ2 test was used to determine the association between *TAGLN2* expression and clinicopathological characteristics. Pearson correlation was used to evaluate the linear relationship between the expression of different genes. The one-way ANOVA test or Student’s *t* test were used for all other data comparisons using GraphPad Prism 6 software. All data are presented as the mean ± standard error. All tests were two-sided, and *P*-values <0.05 were considered to be statistically significant.

## Results

### Increased expression of *TAGLN2* is associated with increasing tumor grade in glioma

**Fig. 1:**
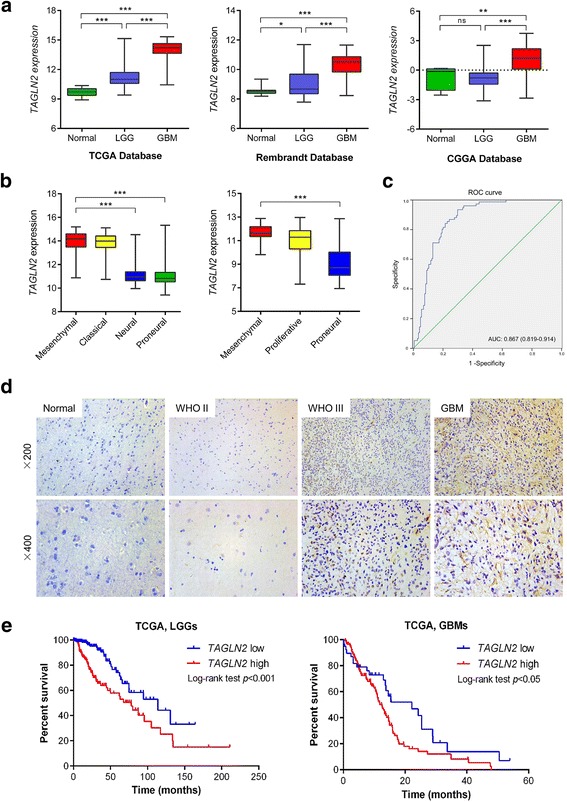
Expression of *TAGLN2* is associated with tumor grade in gliomas. **a** Quantification of *TAGLN2* mRNA expression levels in gliomas in TCGA, Rembrandt, and CGGA datasets. **b** Quantification of GBM subtype-specific *TAGLN2* expression in TCGA and GSE4271 datasets. Log_2_-transformed expression of *TAGLN2* mRNA levels are listed on the Y-axis. Error bars represents the SEM. **c** ROC curve showing sensitivity of TAGLN2 as a marker to distinguish mesenchymal subtype GBM from non-mesenchymal subtype GBM patients. **d** Representative images of IHC staining for TAGLN2 in different grade gliomas and normal brain specimens. Magnification: ×200, upper; ×400, lower. **e** The prognostic significance of *TAGLN2* expression in LGG and GBM patients was analyzed in TCGA (*n* = 667) database. The cut-off level was set at the median value of the *TAGLN2* levels. *P < 0.05; **P < 0.01; ***P < 0.001

To begin to define the function of TAGLN2 in glioma development, gene expression levels of *TAGLN2* were analyzed in GBMs and low grade gliomas (LGGs) as well as normal brain tissues from the TCGA dataset. *TAGLN2* mRNA levels were significantly increased in GBMs compared to LGGs and normal brain tissues in TCGA (*P* < 0.001; Fig. 1a). The expression of *TAGLN2* was also up-regulated in LGGs relative to normal brain (*P* < 0.001). We validated these findings in 3 additional published datasets, Rembrandt, CGGA, and Gravendeel (Fig. 1a; Additional file 1: Fig. S1A). In these datasets, *TAGLN2* was also highly expressed in GBM samples compared to LGGs and normal tissues. However, no significant differences in *TAGLN2* expression were observed between LGGs and normal tissues in CGGA and Gravendeel databases.

Expression profiles have been used to classify gliomas into four distinct molecular subtypes: classical, mesenchymal, neural, and proneural. The mesenchymal subtype has been associated with worse prognosis in patients compared to the proneural subtype [13]. Expression levels of *TAGLN2* were therefore examined on the basis of molecular subtype. *TAGLN2* was high in the mesenchymal subtype in TCGA, CGGA and GSE4271 databases and low in the proneural subtype (Table 1; Fig. 1b; Additional file 1: Fig. S1B). Meanwhile, GSEA analysis showed that mesenchymal related gene signatures were significantly enriched in high TAGLN2 expression samples (Additional file 2: Fig. S2), and ROC curve further showing sensitivity of TAGLN2 as a marker to distinguish mesenchymal subtype from non-mesenchymal subtype GBM patients (Fig. 1c). Protein levels of TAGLN2 were also examined by immunohistochemistry in an independent cohort of human gliomas (*n* = 46) and normal brain tissues (*n* = 5) from Qilu Hospital (Jinan, China). Consistent with the results in mRNA microarrays, TAGLN2 protein was higher in GBMs than LGGs or normal brain (Fig. 1d). Thus, TAGLN2 was positively correlated with increasing tumor grade both in the publicly available databases and in our cohort of primary tumor specimens.

**Table 1:** Correlation of TAGLN2 expression in human glioma patients with different clinicopathological features. *P* values were determined by the Chi-square and Fisher’s exact tests

Variable		TAGLN2 high expression	TAGLN2 low expression	*p* value
Age	≥45	234	91	<0.001
	<45	78	203	
Gender	Male	183	170	0.836
	Female	129	124	
KPS	≥80	154	151	0.005
	<80	46	20	
WHO grade	II	49	165	<0.001
	III	113	126	
	IV	148	4	
TCGA subtype	Neural	34	77	<0.001
	Proneural	65	173	
	Classical	84	2	
	Mesenchymal	93	3	
IDH1 status	Mutant	103	324	<0.001
	Wild-type	225	8	
MGMT promotor	Methylated	171	304	<0.001
	Unmethylated	132	29	
1p/19q	Codeletion	38	132	<0.001
	Non-codeletion	290	202	
TERT expression	Not expressed	113	203	<0.001
	Expressed	221	130	
ATRX status	Mutant	49	146	<0.001
	Wild-type	276	186	

Clinicopathological characteristics of patients in TCGA were also associated with high or low expression of *TAGLN2*. High expression of *TAGLN2* (median value) was statistically associated with patient age (≥ 45 y; *P* < 0.001) and KPS (< 80; *P* = 0.005; Table 1). Some molecular genetic features including *IDH1/2* mutation, MGMT promotor methylation, codeletion of 1p/19q, TERT loss, and *ATRX* mutation have been reported to be associated with favorable prognosis in gliomas [14, 15]. We therefore analyzed whether *TAGLN2* expression correlated with these characteristics. Patients with wild type *IDH1* exhibited higher expression of TAGLN2 than those with mutated *IDH1*. Low *TAGLN2* was also associated with other molecular characteristics, including methylated *MGMT*, 1p/19q codeletion, loss of *TERT* and mutated *ATRX* in tumors (*P* < 0.001; respectively).

### *TAGLN2* expression is associated with poorer patient survival

The prognostic value of *TAGLN2* expression in overall survival (OS) of glioma patients was examined in Kaplan-Meier survival curves. High *TAGLN2* expression (> median value) had a significantly worse prognosis than those with low *TAGLN2* expression in LGG (78.2 vs 114.0 months, *P* < 0.001) and GBM patients (12.5 vs 22.2 months, *P* < 0.05) in TCGA (*n* = 667) (Fig. 1e). These results were validated in Rembrandt (*n* = 329), CGGA (*n* = 302) and Gravendeel (*n* = 284) cohorts (Additional file 1: Fig. S1C), confirming that high *TAGLN2* was statistically related to shorter OS. Furthermore, *TAGLN2* expression was validated as an independent indicator of OS after multivariate Cox regression analysis (HR = 1.713, 95% CI = 1.384 to 2.119, *P* < 0.0001; Table 2). TAGLN2 might therefore be a novel prognostic biomarker in gliomas.

**Table 2:** Univariate and multivariate Cox regression of TAGLN2 expression for overall survival in glioma patients

Variable	Univariate Cox Regression		Multivariate Cox Regression	
	HR (95% CI)	*p*	HR (95% CI)	*p*
Age Increasing years	1.038 (1.023–1.054)	<0.001	0.993 (0.975–1.011)	0.446
Gender Female vs male	0.847 (0.600–1.195)	0.345		
WHO grade High- vs low-	5.840 (4.015–8.494)	<0.001	3.128 (1.969–4.971)	<0.001
TAGLN2 expression High vs low	2.121 (1.843–2.442)	<0.001	1.713 (1.384–2.119)	<0.001
IDH1 status Mutation vs wild-type	0.256 (0.178–0.368)	<0.001	0.869 (0.501–1.510)	0.619
Radiotherapy Yes vs no	0.429 (0.296–0.622)	<0.001	0.439 (0.303–0.638)	<0.001

### Pathway analysis of *TAGLN2* and co-regulated genes

**Fig. 2:**
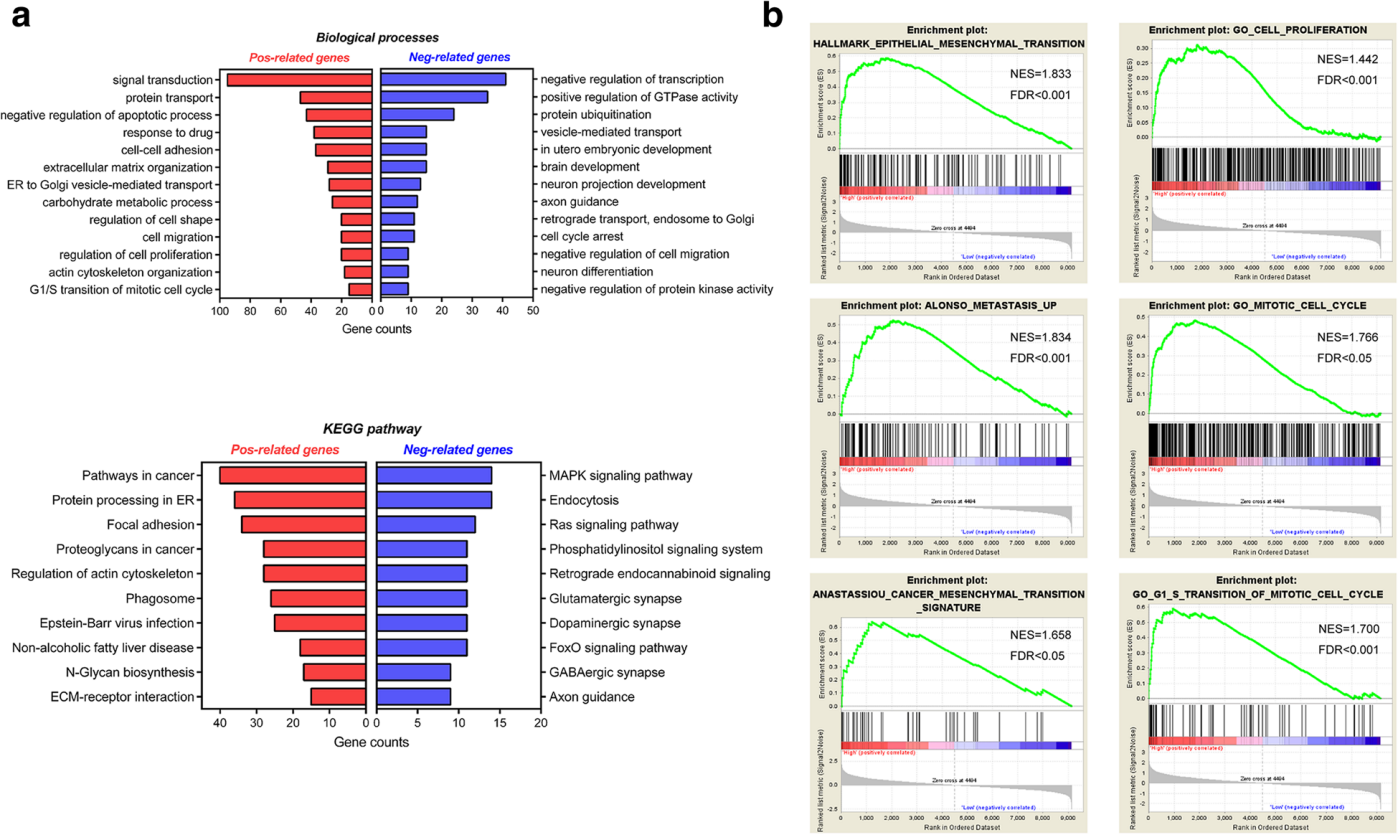
Pathway analysis of TAGLN2 and co-regulated genes. **a** Correlation analysis using TCGA data showing positively (*n* = 1366) and negatively (*n* = 822) correlated genes with *TAGLN2* mRNA expression in human gliomas. Biological processes and KEGG pathway analysis of the positively and negatively correlated genes are illustrated. Potential functions and pathways are listed on the Y-axis. **b** GSEA highlighting positive association of increased *TAGLN2* expression levels with EMT, cancer metastasis, cell proliferation, and G1-S phase transition of cell cycle progression. FDR = false discovery rate; NES = normalized enrichment score

To further explore potential biological functions of TAGLN2 in gliomas, correlation analysis of *TAGLN2* expression in whole-genome profiling was performed. Datasets were analyzed for *TAGLN2* positive-related (*n* = 1366) and negative-related genes (*n* = 822), which were subsequently subjected to pathway analysis (Additional file 5: Table S1; *P* < 0.01). Gene ontology (GO) analysis indicated that *TAGLN2* positively associated genes were most involved in malignant process, including cell adhesion, extracellular matrix organization, cell proliferation, as well as cell cycle transition; On the contrary, *TAGLN2* negatively related genes were mainly enriched in relatively differentiated cellular process or protective functions, such as brain development, neuron differentiation, cell cycle arrest, and negative regulation of cell motility (Fig. 2a). KEGG analysis demonstrated that *TAGLN2* was enriched in pathways in cancer, focal adhesion and regulation of the actin cytoskeleton. Finally, GSEA analysis revealed that high levels of *TAGLN2* were significantly related to EMT, cancer metastasis, and the G1-S phase transition of cell cycle progression (Fig. 2b). Altogether, these data further implicated the malignant property of TAGLN2 in gliomagenesis.

### *TAGLN2* silencing reduces invasion and inhibits mesenchymal properties in glioma cells in vitro

**Fig. 3:**
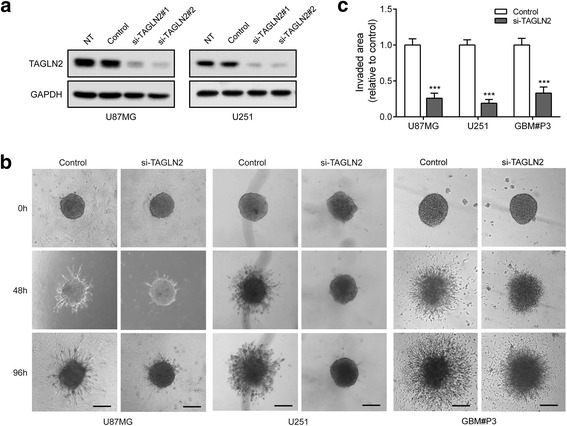
*TAGLN2* knockdown decreases invasive ability of glioma cells. **a** Western blot of lysates (20 μg) from U87MG and U251 cells transfected with TAGLN2 and control siRNAs incubated with TAGLN2 antibody. GAPDH was used as a loading control. **b** Representative images of invaded spheroids in 3D invasion assay for U87MG, U251 and GBM#P3 cells transfected with TAGLN2 and control siRNAs evaluated at 48 h and 96 h are shown. Scale bar = 200 mm. **c** The area covered by invading cells quantitated after 96 h. NT: no treatment; Control: non-silencing siRNA; si-TAGLN2: siRNAs targeting *TAGLN2*

Based on the pathway analysis, we first assessed the role of TAGLN2 in cell motility and invasion using a 3D collagen spheroid invasion assay. Two independent siRNA sequences against *TAGLN2* were designed. Both demonstrated efficient silencing of TAGLN2 in two malignant glioma cell lines with high invasive potential, U87MG and U251 (Fig. 3a). Silencing *TAGLN2* with a single siRNA reduced the area invaded by U87MG, U251, as well as primary GBM#P3 spheroids relative to controls, respectively (Fig. 3b and c).

**Fig. 4:**
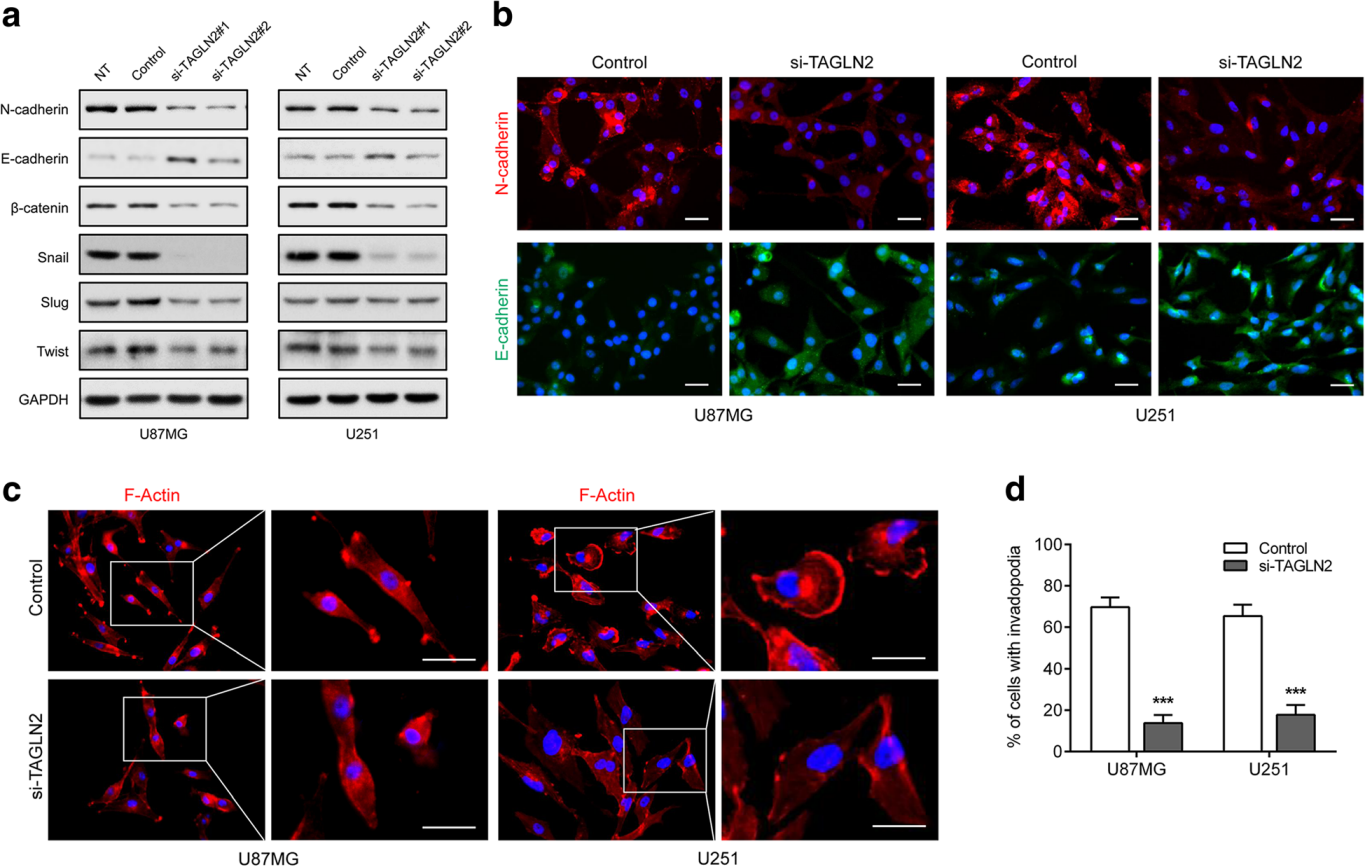
*TAGLN2* knockdown inhibits mesenchymal transition and reduces formation of invadopodia in glioma cells. **a** Western blot for protein levels of EMT components in lysates (20 μg) from U87MG and U251 cells transfected with siRNA against *TAGLN2* and controls. GAPDH was used as a loading control. **b** Immunofluorescence images of N-cadherin (red), E-cadherin (green), and nuclear (blue) staining in U87MG and U251 at 48 h after siRNA transfection. **c** Immunofluorescence images for F-actin (red) and nuclear (blue) staining in U87MG and U251 at 48 h after siRNA transfection. Scale bar = 30 μm. **d** Invadopodia formation was quantified as a percentage of the cells with invadopodia on 30 randomly selected cells in each group. Results are representative of three independent experiments. NT: no treatment; Control: nonsilencing siRNA; si-TAGLN2: siRNAs targeting *TAGLN2*

It is well known that the epithelial–mesenchymal transition (EMT) process plays an essential role in the invasiveness and metastasis of various cancers [16, 17]. We therefore investigated whether TAGLN2 regulated EMT in glioma cells. Western blot analysis revealed that knockdown of TAGLN2 led to decreases in several mesenchymal factors, including N-cadherin, β-catenin, Snail, Slug, and Twist, and significant increases in the epithelial marker (E-cadherin) compared to controls (Fig. 4a and b). Moreover, fluorescence staining demonstrated that TAGLN2 knockdown decreased the formation of cell invadopodia (Fig. 4c and d), the F-actin-rich leading edge of invading cells, which is a key structure in cancer invasion [18]. Thus, TAGLN2 may contribute to invasiveness in glioma cells by promoting EMT and the formation of invadopodia in glioma cell lines.

Meanwhile, the importance of TAGLN2 in regulating mesenchymal characteristics in primary GBM cells was detected. We found that knocking down of TAGLN2 lead to the suppression of neurosphere formation ability and cell growth of GBM#P3 cells, accompanied with the reduction of key mesenchymal markers, including CD44, CHI3L1, N-cadherin and β-catenin (Additional file 3: Fig. S3). Altogether, these results suggest that TAGLN2 plays a role in the maintenance of the mesenchymal signature of GBM.

### Knockdown of *TAGLN2* induces cell cycle arrest and apoptosis in human glioma cells

**Fig. 5:**
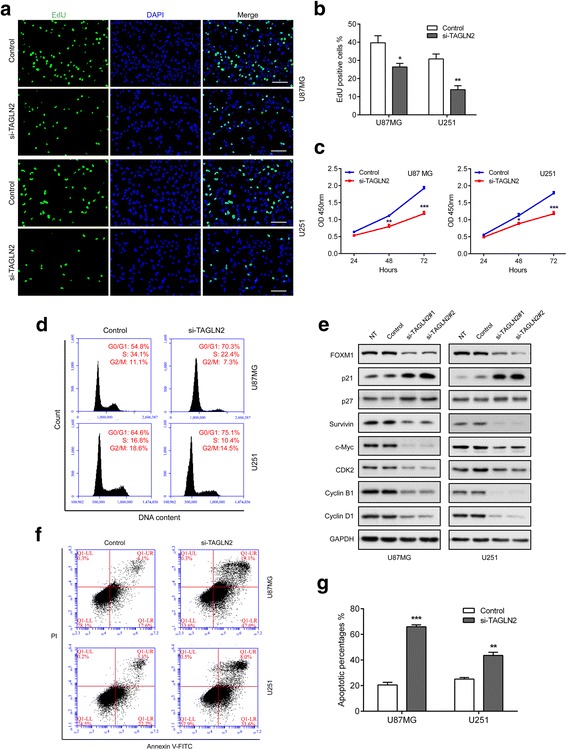
*TAGLN2* knockdown inhibits cell proliferation and induces cell cycle arrest. U87MG and U251 cells transfected with TAGLN2 siRNA or controls and characterized in the following assays: (**a**, **b**) EdU performed 48 h after transfection (scale bar = 100 μm); (**c**) growth curve based on OD450 using the CCK-8 assay; (**d**) cell cycle profiles determined from PI staining in flow cytometry; (**e**) Western blot to detect expression levels of the known cell cycle regulatory factors indicated. GAPDH was used as a loading control; (**f**, **g**) % apoptosis as determined with Annexin V-FITC antibody and PI staining in flow cytometry. Data are shown as the mean ± SEM from three independent experiments. *P < 0.05; **P < 0.01; ***P < 0.001, relative to control. NT: no treatment; Control: non-silencing siRNA; si-TAGLN2: siRNAs targeting *TAGLN2*

The results of our pathway analysis implicated a possible role for TAGLN2 in regulating cell proliferation, the cell cycle, and apoptosis. To directly test the role of TAGLN2 in glioma cell survival and proliferation, cells were transfected with siRNA to knock down *TAGLN2*, and EdU as well as CCK-8 assays were performed. Down-regulation of TAGLN2 resulted in a statistically significant decrease in OD450 values as well as the percentage of EdU positive cells in both U87MG and U251 48 h after transfection (*P* < 0.05 and *P* < 0.01, respectively; Fig. 5a and c). Cell cycle analysis also demonstrated that knockdown of *TAGLN2* increased the population of the U87MG and U251 cells in the G0/G1 phase by ~ 15.5% and 10.5%, respectively (Fig. 5d). Futhermore, *TAGLN2* silencing promoted apoptotic cell death by ~ three- and two-fold compared with controls in U87MG and U251 cell lines, respectively (*P* < 0.001 and *P* < 0.01, respectively; Fig. 5f and g).

Next, we investigated the downstream targets of TAGLN2 using western blot. TAGLN2 knockdown significantly decreased the level of Fork head box M1 (FoxM1), an oncogenic transcription factor essential for cancer progression in various types of cancers including gliomas (Fig. 5e) [19, 20]. In addition, cyclin-dependent kinase 2 (CDK2), cyclin B1, and cyclin D1 expression were decreased after TAGLN2 silencing. In contrast, cyclin-dependent kinase inhibitors, including p21 and p27 which were identified as tumor suppressors, were increased in the si-TAGLN2 group [21, 22]. Furthermore, a marked reduction in c-Myc and Survivin, downstream targets of p21, were observed after TAGLN2 knockdown. Taken together, these results indicated that loss of TAGLN2 suppressed cell cycle progression and induced apoptosis in glioma cells.

### TGFβ2 induces TAGLN2 in glioma cell lines

**Fig. 6:**
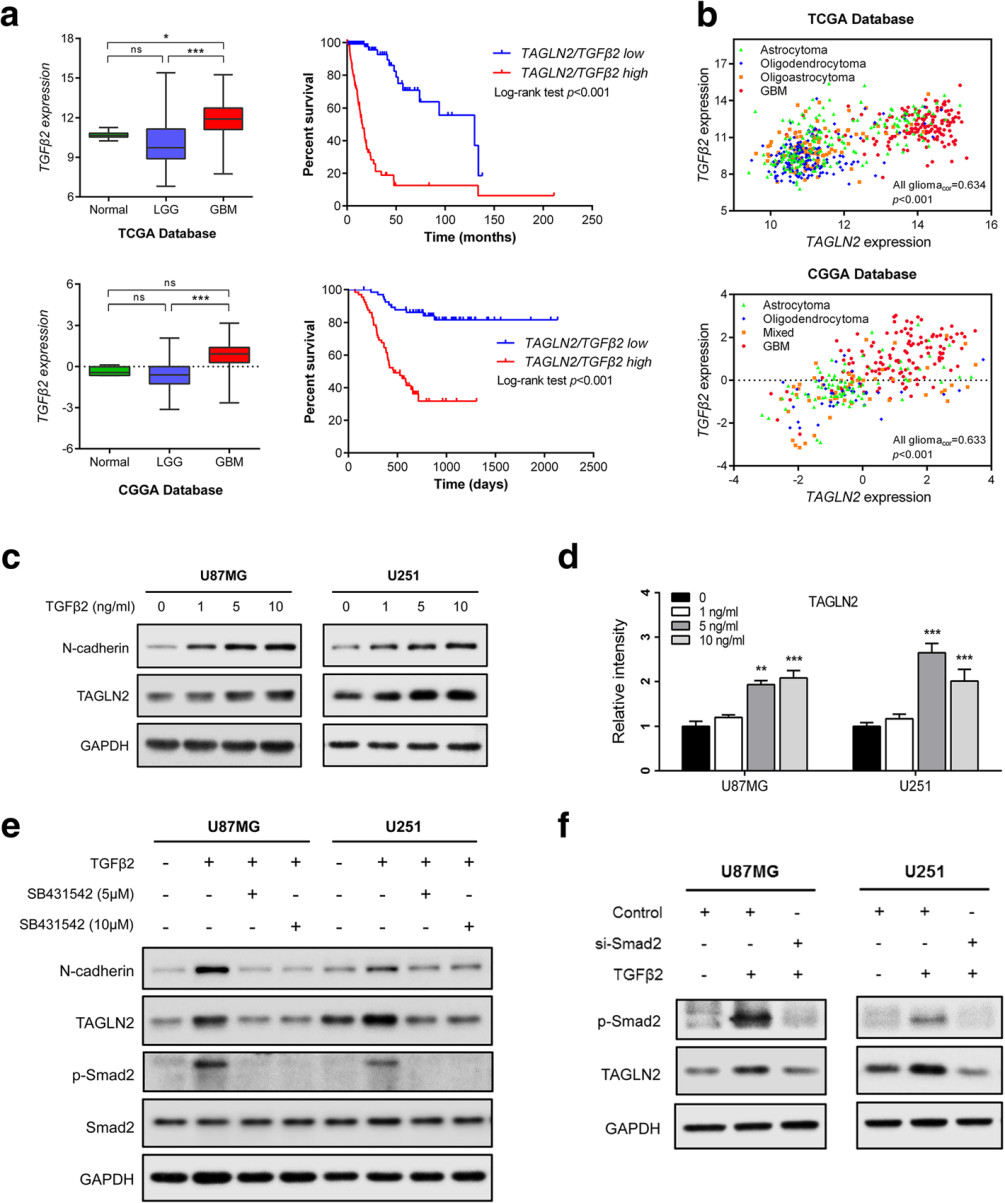
TGFβ stimulates TAGLN2 protein expression in gliomas. **a** Quantification of *TGFβ2* mRNA expression levels in glioma patients in TCGA and CGGA datasets, and survival of patients with low/high coexpression of *TAGLN2*/*TGFβ2*. **b** Correlation between *TAGLN2* and *TGFβ2* in gliomas determined using TCGA (left panel) and CGGA (right panel) datasets. The statistical significance of correlation was evaluated using a linear regression model (TCGA all glioma_cor_ = 0.634, *P* < 0.001; CGGA all glioma_cor_ = 0.633, *P* < 0.001). **c**, **d** Western blot for TAGLN2 in cells treated with different concentrations of TGFβ2 (0, 1, 5 and 10 ng/mL) for 48 h. **e** Western blot for TAGLN2, N-cadherin, p-Smad2 and Smad2 in U87MG and U251 cells treated with TGFβ2 with/without SB431542 (5 and 10 μM). **f** Western blot for TAGLN2, p-Smad2 in U87MG and U251 cells transfected with Smad2 siRNA or controls. Results are representative of three independent experiments. *P < 0.05; **P < 0.01; ***P < 0.001

How TAGLN2 is regulated might provide insight into additional pathways that can ultimately be exploited for therapeutic treatment. Activation of transforming growth factor beta (TGFβ)/Smad signaling, for example, has been found to induce *TAGLN* expression in human bone marrow-derived stromal stem cells (hMSC) [23]. In addition, TGFβ2 is a TGFβ family member that has been found to be specifically involved in brain tumor development and progression [24, 25]. In fact, in both TCGA and CGGA datasets, *TGFβ2* was higher in GBM than in LGG, just as for *TAGLN2*, and patients with high expression of both *TGFβ2* and *TAGLN2* exhibited significantly poorer OS (*P* < 0.001, respectively; Fig. 6a). Furthermore, analysis of the correlation between *TGFβ2* and *TAGLN2* revealed a statistically linear relationship between the two mRNAs (TCGA all glioma_cor_ = 0.634, *P* < 0.001; CGGA all glioma_cor_ = 0.633, *P* < 0.001; Fig. 6b). We therefore examined by Western blot whether TGFβ2 might be a growth factor stimulating TAGLN2 protein levels in cells in vitro. Addition of TGFβ2 (5 ng/mL) to cell culture led to increases in TAGLN2 protein levels by ~ two- and three-fold in U87MG and U251 cell lines, respectively (Fig. 6c and d). In contrast, TAGLN2 was decreased upon exposure of cells to the TGFβ/Smad pathway specific inhibitor SB431542 (5 μM and 10 μM; Fig. 6e). To further clarify whether TGFβ2 regulates TAGLN2 through Smad or Smad-independent pathways, cells were transfected with Smad2-siRNA. In the presence of TGFβ2, TAGLN2 expression was decreased in Smad2 knockdown cells compared with control group (Fig. 6f). Thus, these results indicated that TAGLN2 expression induced by TGFβ2 was Smad-dependent in gliomas.

### *TAGLN2* silencing inhibits tumorigenesis in vivo

**Fig. 7:**
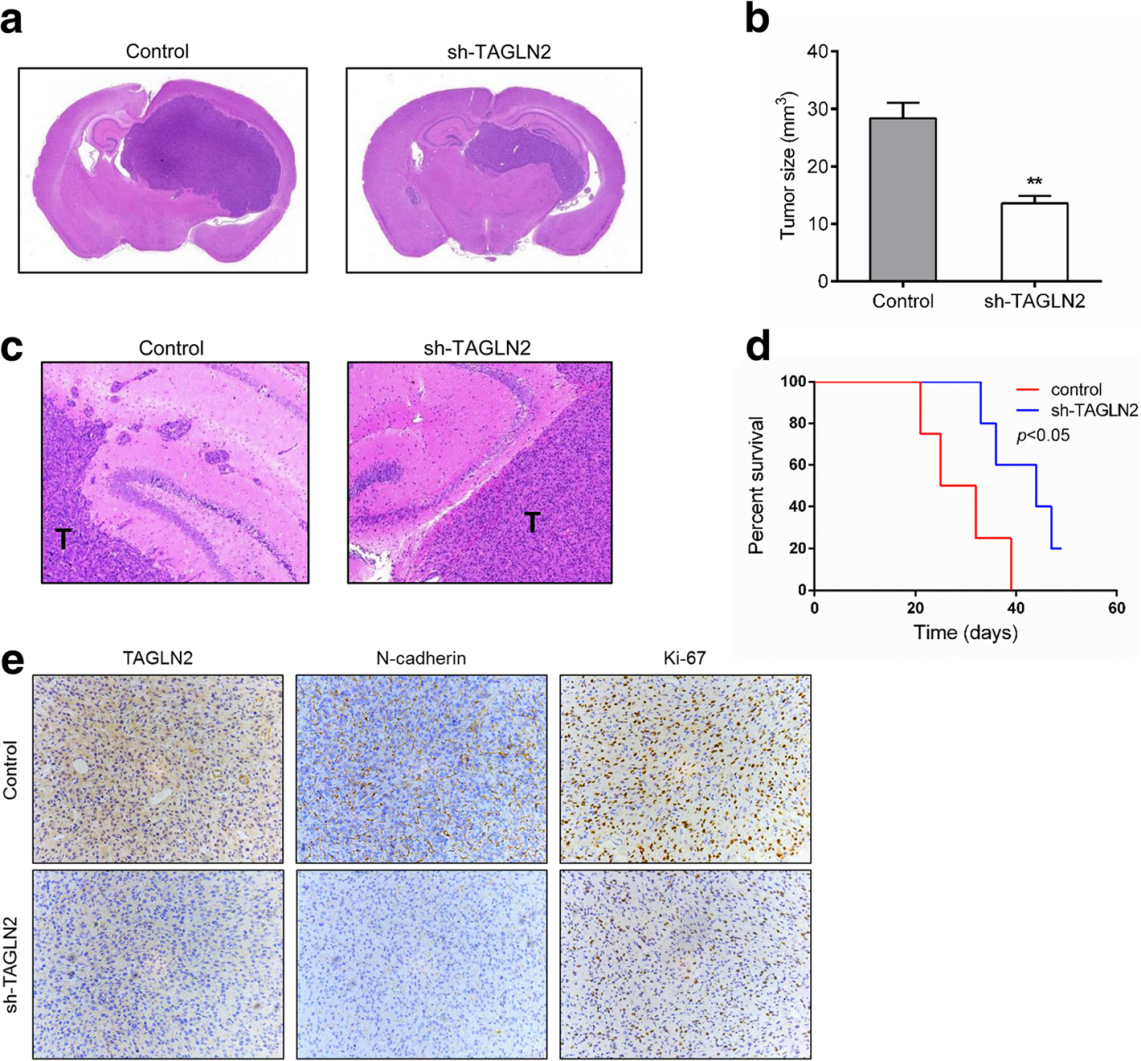
*TAGLN2* silencing inhibited tumorigenesis in vivo. **a** H&E staining of sections from mouse brains with U87MG control or sh-TAGLN2 xenografts at ~ 4 weeks after implantation with 1 × 10^6^ cells, and (**b**) tumor size (mm^3^) was measured. **c** H&E staining of sections at tumor margins in intracranial U251 control or sh-TAGLN2 xenografts. **d** Survival analysis for animals implanted with U87MG sh-TAGLN2 or control cells (*P* < 0.05 by log-rank test; *n* = 5 animals per group) (**e**) IHC for TAGLN2, N-cadherin and Ki-67 in sections from indicated xenografts. Magnification: ×200

To further examine the function of TAGLN2 protein in human gliomas, growth of cells depleted of the protein was assessed in vivo. Glioma cells were transduced with lentiviral constructs expressing TAGLN2-targeting shRNA or control shRNA. Orthotopic xenografts were established by implanting sh-TAGLN2 or control cells into nude mice. Animals bearing sh-TAGLN2 cells displayed significantly reduced tumor size (Fig. 7a and b), more circumscribed borders (Fig. 7c) and increased survival relative to controls (44.0 vs 28.5 days, *P* < 0.05; Fig. 7d). Immunohistochemistry confirmed that TAGLN2 protein levels were reduced in xenografts generated with sh-TAGLN2 cells. The EMT classical marker N-cadherin and the proliferation index marker Ki-67 were also decreased in sh-TAGLN2 xenografts (Fig. 7e). These results demonstrated that *TAGLN2* knockdown led to reduced growth and invasion of glioma cells in vivo. Furthermore, we also explore the intratumor heterogeneity of TAGLN2 expression via IVY GAP Atlas (http://ivygap.org/), and the in situ hybridization (ISH) results indicated the positive staining of TAGLN2 both in tumor center and infiltrating zone in GBM tissue (Additional file 4: Fig. S4).

## Discussion

Human gliomas have been well characterized molecularly. Yet the significance of many of the changes in gene expression have not been attributed to any underlying biological function. Here, we investigated the function of TAGLN2, a gene that was found to be highly expressed in GBMs compared to LGGs. High *TAGLN2* expression was associated with the mesenchymal molecular phenotype in human gliomas and thus poor prognosis in glioma patients. In contrast, low *TAGLN2* mRNA levels were linked to other positive prognostic markers, including *IDH1* and *ATRX* mutations, methylated *MGMT*, 1p/19q codeletion, and loss of *TERT*. Finally, functional studies using RNA knockdown implicated a role for TAGLN2 in promoting cell invasion and proliferation in human gliomas.

Glioma progression is a dynamic process in which EMT is a key event driving invasion of tumor cells [26, 27]. Several EMT-related factors have been previously associated with increased invasion and poor prognosis in gliomas [19]. Here, we observed that TAGLN2 depletion significantly decreased glioma cell invasiveness and reversed EMT features, including changes in epithelial (E-cadherin) and mesenchymal markers (N-cadherin, Snail, Slug, Twist) in glioma cells. Using immunofluorescence staining, we observed F-actin cytoskeletal changes induced by TAGLN2 depletion in gliomas. *TAGLN2* silencing appeared to specifically suppress the F-actin-rich leading edge in glioma cells, thus reducing the formation of invadopodia during cell invasion. According to the study by Na et al., TAGLN2 blocks actin depolymerization and competes with cofilin to protect F-actin during the formation of immunological synapse in T cells, and knockout of TAGLN2 significantly destabilized F-actin ring formation, resulting in decreased cell adhesion and spreading [28, 29]. Therefore, we hypothesize that TAGLN2 may promote invadopodia formation of glioma cells via competing with cofilin and suppressing actin depolymerization. Altogether, these results indicated that TAGLN2 may serve as a crucial regulator of invasion and aggressiveness by inducing mesenchymal-like properties in gliomas.

Abnormal cell proliferation and growth are hallmark characteristics of human gliomas. Many genetic changes lead to uncontrolled growth through dysregulation of proteins directly involved in cell cycle progression and cell apoptosis [30]. GO and GSEA analysis indicated that TAGLN2 might indeed promote growth through functions in cell cycle progression and cell survival. In vitro and in vivo experiments supported this analysis. *TAGLN2* knockdown in glioma cells induced cell cycle arrest at G0-G1 and cell apoptosis, and reduced growth in orthotopic xenografts. The fact that knockdown of *TAGLN2* leads to reduced cell proliferation in vitro and in vivo renders the gene/pathway as a potential molecular target for therapy. Moreover, to uncover the potential molecular mechanisms by which TAGLN2 promote glioma development, we detect the expression change of FoxM1, an oncogenic transcriptional factor that regulates some key mediators of cell cycle progression, including CDK2, cyclin B1, cyclin D1, p21 and p27 [20]. Meanwhile, recent studies have revealed that the oncogenic potential of FoxM1 is determined by its capacity to transactivate target genes that are implicated in different phases of cancer development [31], including cancer survival, invasiveness, EMT process, and angiogenesis. In addition, a variety of oncogenic genes including Survivin, c-Myc, β-catenin, and Snail were identified to be regulated by FoxM1. In the present study, *TAGLN2* knockdown led to significantly reduced levels of FoxM1, as well as downstream oncogenic factors including CDK2, cyclin B1, and cyclin D1, c-Myc and Survivin. Meanwhile, tumor suppressor p21 and p27 were induced after *TAGLN2* depletion. These data suggest that TAGLN2 activates FoxM1 signaling during gliomagenesis. However, further investigation is necessary to elucidate the mechanism of TAGLN2 regulation of FoxM1 axis in glioma.

Consistent with our current study, TAGLN2 has been reported to be up-regulated and possess proto-oncogenic functions in a variety of cancers. For example, high expression of TAGLN2 in tumor-derived lung cancer endothelial cells was associated with clinical stage, tumor size, and tumor development in lung cancer tissues [10]. In addition, TAGLN2 overexpression has been reported to be correlated to lymph node metastasis and histological neural invasion of bladder [32], colorectal [8], esophageal [33], and gastric [34] cancer. Similarly, the elevated expression of TAGLN2 was observed in uterine cervical squamous cell carcinoma (SCC), while suppression of TAGLN2 in human uterine SCC cells significantly inhibited tumor growth and invasion [35]. However, in gynecological malignancies, decreased expression of TAGLN2 was found in metastatic cells in comparison to primary tumors [36]. Meanwhile, TAGLN2 was suggested to be negatively correlated with breast cancer metastasis, and metastatic breast cancer cell line exhibited down-regulation of TAGLN2 protein [37]. These contradictory results reveal a complex role of TAGLN2 in tumorigenesis, urging further investigation.

Based on its potentially crucial role in promoting glioma development, we were also interested in identifying upstream regulators of TAGLN2 expression. It is well known that TGFβ is a multifunctional cytokine that modulates biological processes, such as cell stemness, angiogenesis, cell growth, and immune function [38, 39]. In addition, studies have shown that TGFβ2 overexpression promotes motility, invasion and EMT in cells from some cancer types [40], thus indicating a role for the TGFβ2/Smad signaling pathway in tumorigenesis. In our study, we observed a strong correlation between *TGFβ2* and *TAGLN2* mRNA levels in cases from TCGA and CGGA databases, and in cell culture, TGFβ2 induced TAGLN2 protein in U87MG and U251 cells. The TGFβ type I receptor specific inhibitor SB431542 prevented TAGLN2 induction, and therefore further supported a role for TGFβ2/Smad signaling in the regulation of TAGLN2 in glioma. However, the precise molecular mechanisms of the cross-talk between TAGLN2 and TGFβ2 signaling in gliomas require further investigation.

## Conclusions

We demonstrated that increased *TAGLN2* expression levels were associated with higher tumor grade in human gliomas, and thus the mesenchymal molecular GBM subtype and unfavorable prognosis. Knockdown experiments highlighted the function of TAGLN2 in promoting glioma cell invasion, the EMT phenotype, and tumor growth. TAGLN2 may therefore serve as a novel biomarker and a potential therapeutic target in the treatment of human GBM.

## Additional files

Additional file 1: Fig. S1. The prognostic values of TAGLN2 in validated cohorts. (A) Quantification of *TAGLN2* mRNA expression levels in gliomas in Gravendeel datasets. (B) Quantification of GBM subtype-specific *TAGLN2* expression in CGGA datasets. Log_2_-transformed expression of *TAGLN2* mRNA levels are listed on the Y-axis. Error bars represents the SEM. (C) The prognostic significance of *TAGLN2* expression in LGG and GBM patients was analyzed in Rembrandt (*n* = 329), CGGA (*n* = 302) and Gravendeel (*n* = 284) databases. The cut-off level was set at the median value of the *TAGLN2* levels. ***P < 0.001. (TIFF 4566 kb)

Additional file 2: Fig. S2. TAGLN2 enrichment in mesenchymal subtype gliomas. (A) GSEA analysis showed that mesenchymal-associated genes were significantly enriched in HGG compared to LGG. (B and C) In TCGA data, TAGLN2 mRNA levels were positively correlated with mesenchymal markers (CD44, CHI3L1, POSTN, FoxM1, IL6, and STAT3), but negatively correlated with proneural markers (OLIG2, NF1, PDGFRA, SOX4, NOTCH1, and DLL3). (TIFF 1773 kb)

Additional file 3: Fig. S3. TAGLN2 regulates mesenchymal markers in primary GBM cells. (A) Knocking down TAGLN2 in mesenchymal GBM#P3 cells significantly reduced the expression of mesenchymal markers including CD44, CHI3L1, N-cadherin and FoxM1. GAPDH was used as a loading control. (B and C) Neurosphere formation capacity of GBM#P3 cells decreased significantly after TAGLN2 knockdown (scale bar = 100 μm). Results are representative of three independent experiments. *P < 0.05. (TIFF 801 kb)

Additional file 4: Fig. S4. TAGLN2 expression pattern in different anatomical structures in GBM tissue. Representative images of hematoxylin-eosin (H&E) staining, in situ hybridization (ISH) for TAGLN2 and anatomic features of one GBM sample. (TIFF 2063 kb)

Additional file 5: Table S1. List of TAGLN2-related genes. (XLSX 38 kb)

## Abbreviations

CDK2: Cyclin-dependent kinase 2
EMT: Epithelial-mesenchymal transition
FoxM1: Fork head box M1
GBM: Glioblastoma multiforme
GO: Gene ontology
GSEA: Gene set enrichment analysis
IHC: Immunohistochemistry
KEGG: Kyoto Encyclopedia of Genes and Genomes
OS: Overall survival
TAGLN2: Transgelin-2
TGFβ: Transforming growth factor beta
