# Supplementary figures and images for: TAGLN2 is a candidate prognostic biomarker promoting tumorigenesis in human gliomas
Source: J Exp Clin Cancer Res. 2017 Nov 6;36:155. doi: 10.1186/s13046-017-0619-9 (PMC5674233; doi:10.1186/s13046-017-0619-9)

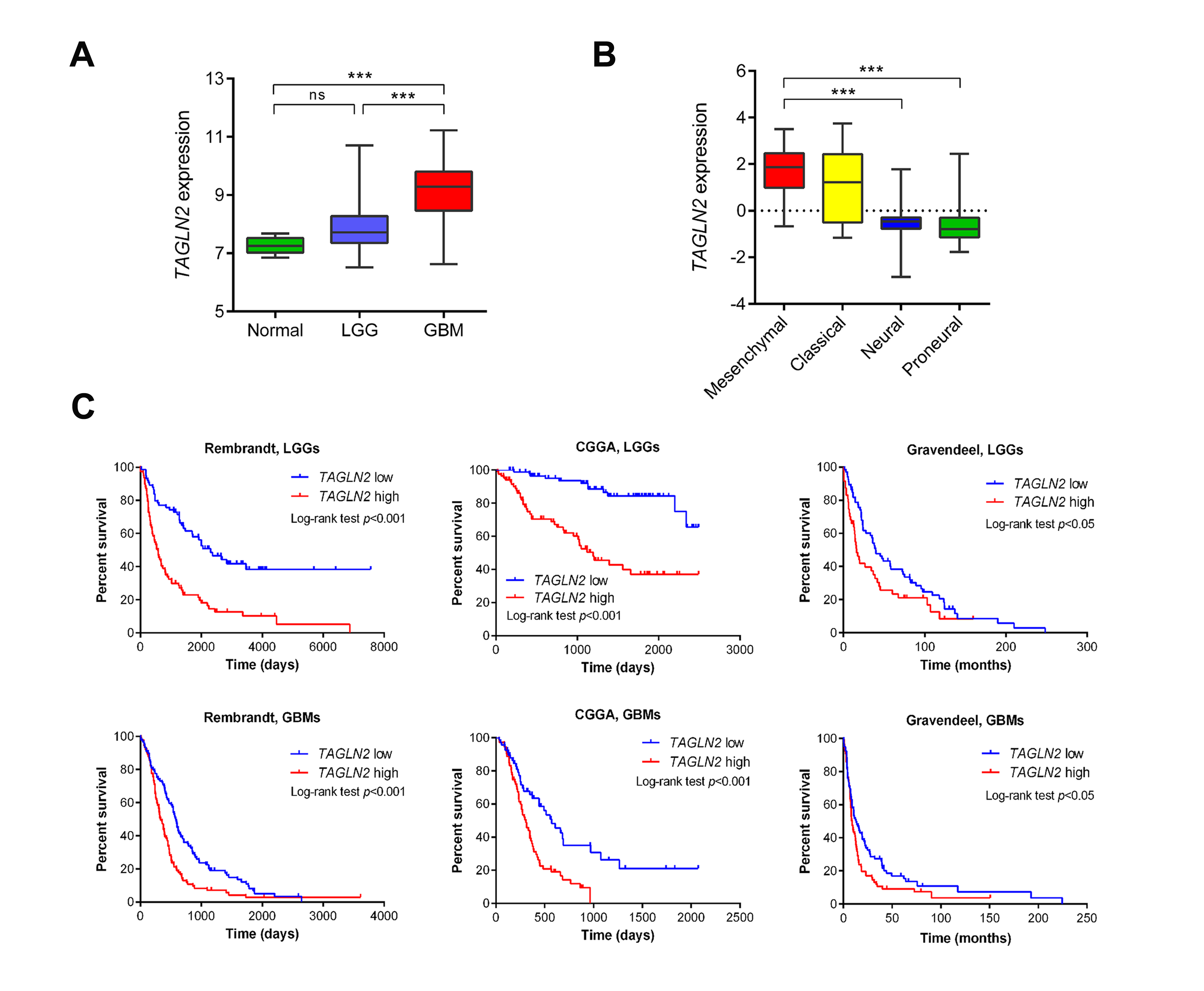

Supplement: Supplementary file 1 — The prognostic values of TAGLN2 in validated cohorts. (A) Quantification of TAGLN2 mRNA expression levels in gliomas in Gravendeel datasets. (B) Quantification of GBM subtype-specific TAGLN2 expression in CGGA datasets. Log2-transformed expression of TAGLN2 mRNA levels are listed on the Y-axis. Error bars represents the SEM. (C) The prognostic significance of TAGLN2 expression in LGG and GBM patients was analyzed in Rembrandt (n = 329), CGGA (n = 302) and Gravendeel (n = 284) databases. The cut-off level was set at the median value of the TAGLN2 levels. ***P < 0.001. (TIFF 4566 kb) [file 13046_2017_619_MOESM1_ESM.tif]

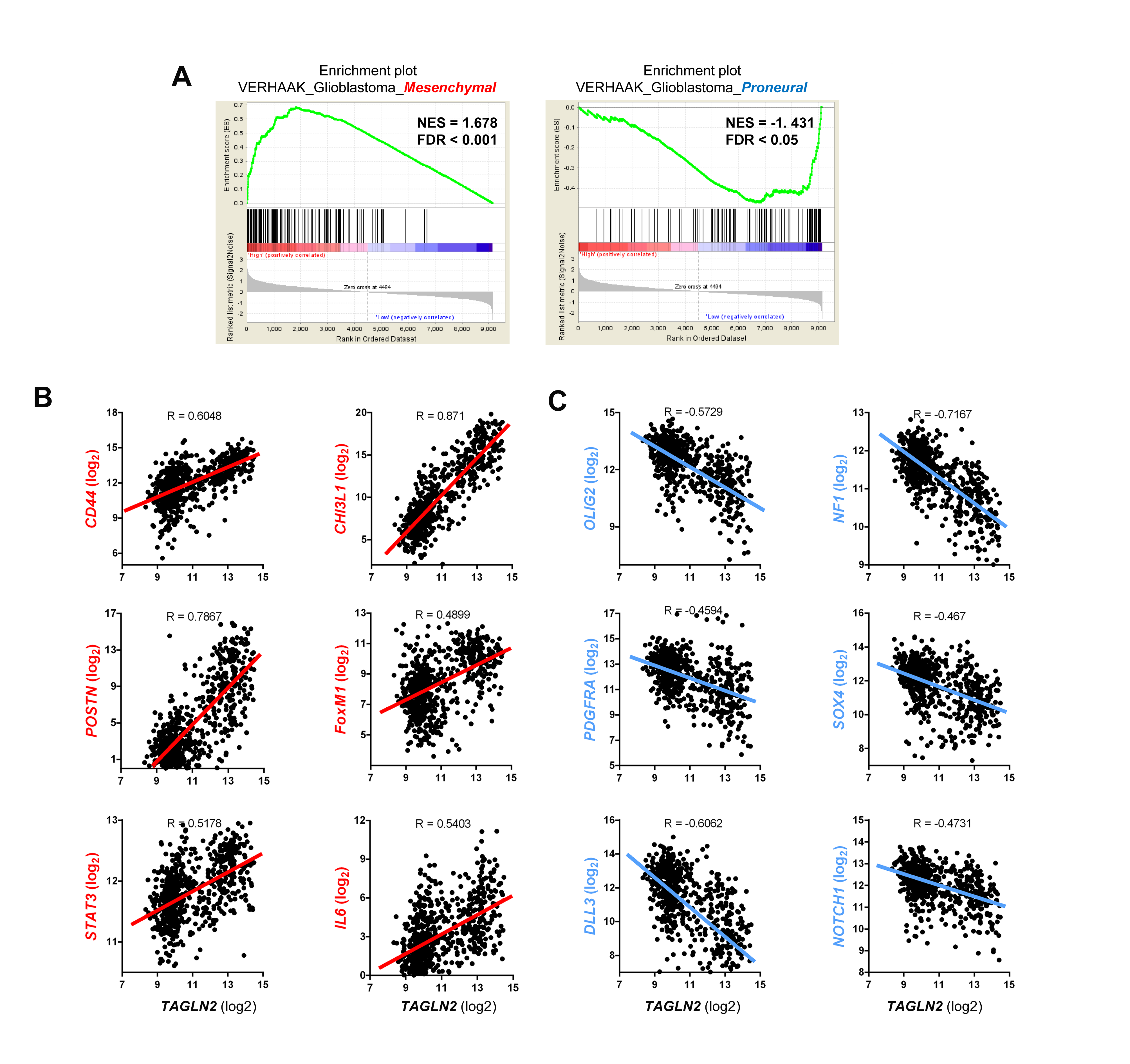

Supplement: Supplementary file 2 — TAGLN2 enrichment in mesenchymal subtype gliomas. (A) GSEA analysis showed that mesenchymal-associated genes were significantly enriched in HGG compared to LGG. (B and C) In TCGA data, TAGLN2 mRNA levels were positively correlated with mesenchymal markers (CD44, CHI3L1, POSTN, FoxM1, IL6, and STAT3), but negatively correlated with proneural markers (OLIG2, NF1, PDGFRA, SOX4, NOTCH1, and DLL3). (TIFF 1773 kb) [file 13046_2017_619_MOESM2_ESM.tif]

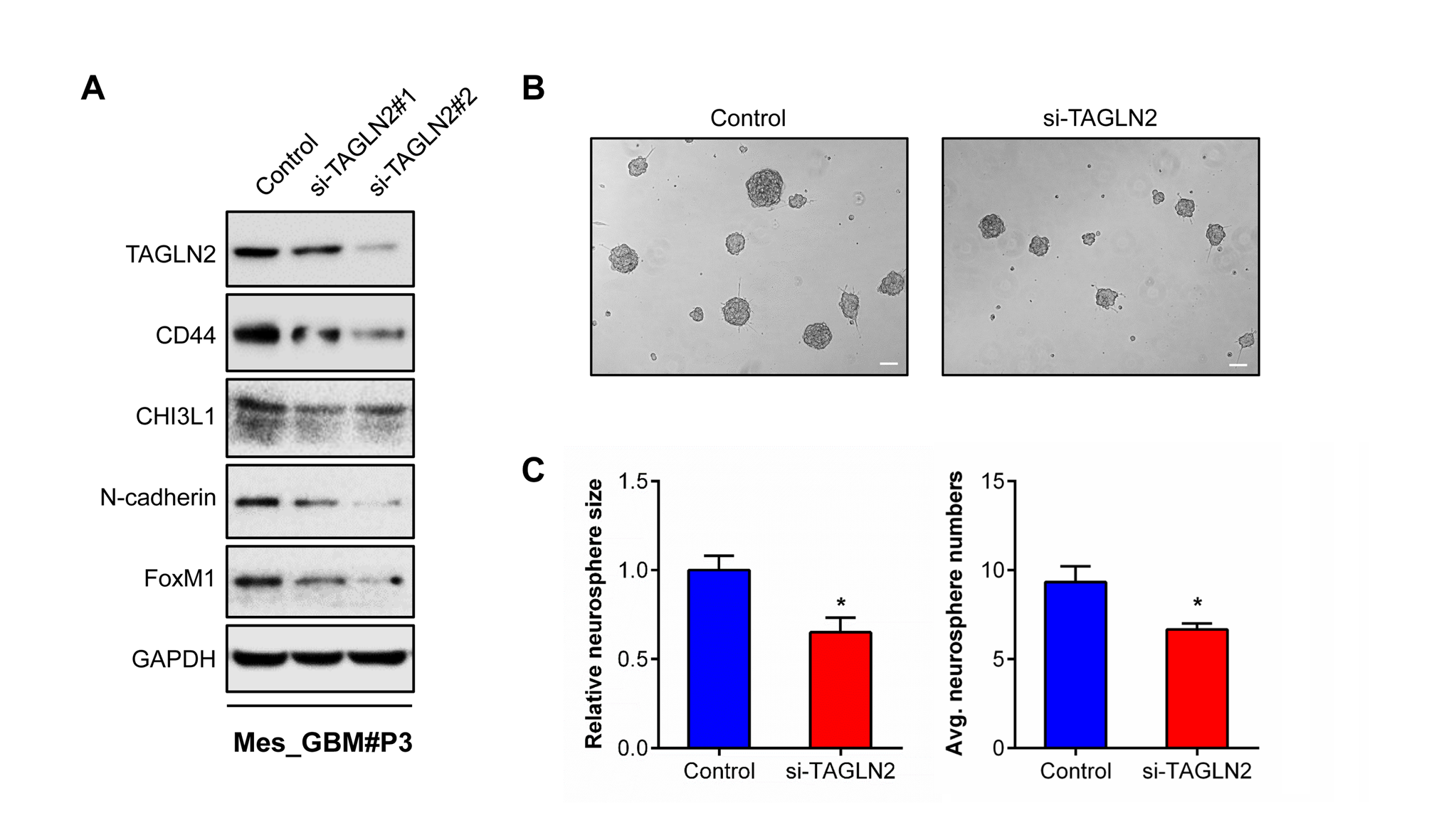

Supplement: Supplementary file 3 — TAGLN2 regulates mesenchymal markers in primary GBM cells. (A) Knocking down TAGLN2 in mesenchymal GBM#P3 cells significantly reduced the expression of mesenchymal markers including CD44, CHI3L1, N-cadherin and FoxM1. GAPDH was used as a loading control. (B and C) Neurosphere formation capacity of GBM#P3 cells decreased significantly after TAGLN2 knockdown (scale bar = 100 μm). Results are representative of three independent experiments. *P < 0.05. (TIFF 801 kb) [file 13046_2017_619_MOESM3_ESM.tif]

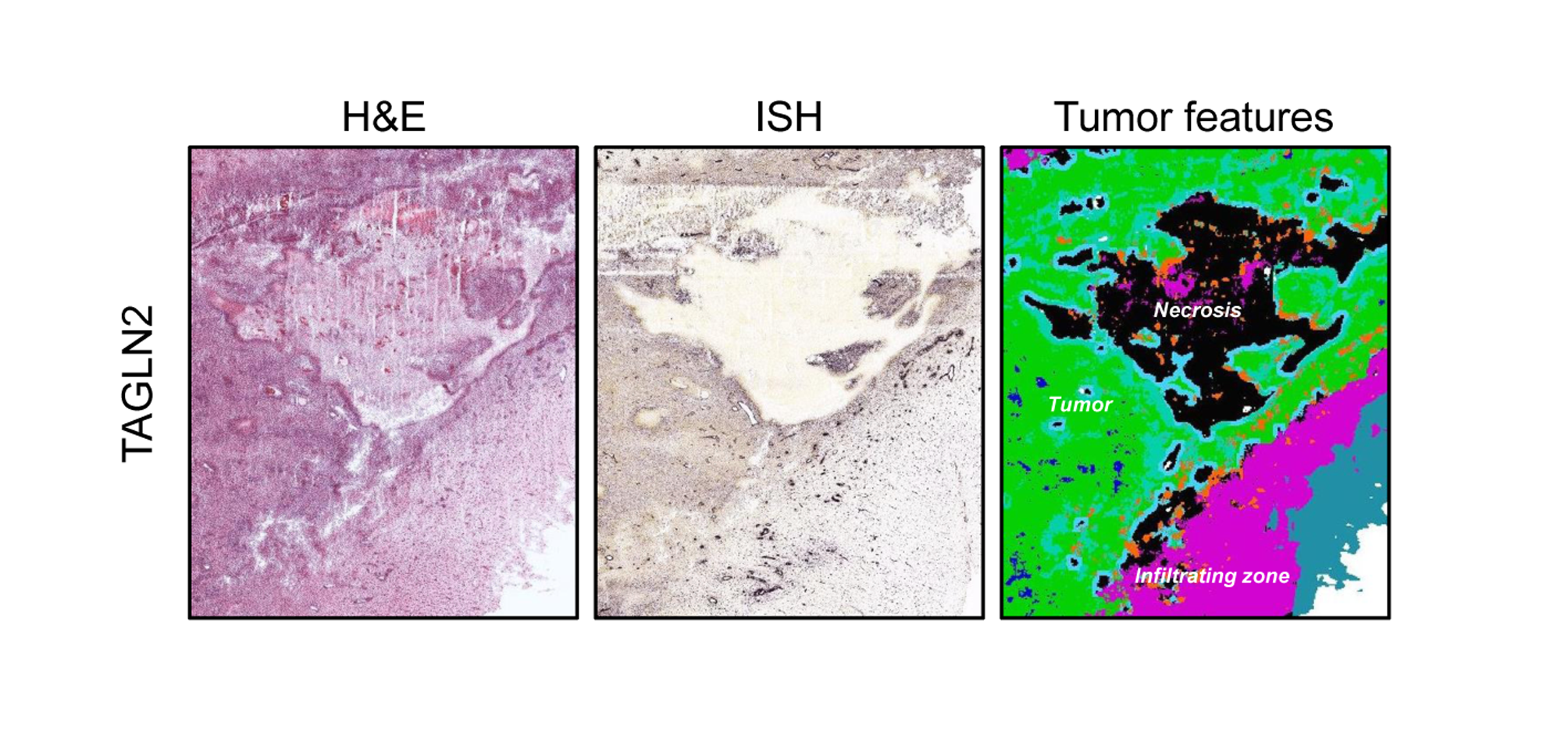

Supplement: Supplementary file 4 — TAGLN2 expression pattern in different anatomical structures in GBM tissue. Representative images of hematoxylin-eosin (H&E) staining, in situ hybridization (ISH) for TAGLN2 and anatomic features of one GBM sample. (TIFF 2063 kb) [file 13046_2017_619_MOESM4_ESM.tif]
